# Evaluating the quality of routinely reported data on malaria commodity stocks in Guinea, 2014–2016

**DOI:** 10.1186/s12936-018-2603-z

**Published:** 2018-12-07

**Authors:** Yu Sun, Timothée Guilavogui, Alioune Camara, Mohamed Dioubaté, Babacar Deen Toure, Claude Bahati, Marie Paule Fargier, Jessica Butts, Patrick Condo, Abdoulaye Sarr, Mateusz M. Plucinski

**Affiliations:** 1 Rollins School of Public Health, Emory University, Atlanta, GA USA; 2 National Malaria Control Program, Conakry, Guinea; 3 Systems for Improved Access to Pharmaceuticals and Services, Conakry, Guinea; 4 Malaria Branch, Division of Parasitic Diseases and Malaria, Centers for Disease Control and Prevention, Atlanta, GA USA; 5 U.S. President’s Malaria Initiative, Centers for Disease Control and Prevention, Atlanta, GA USA; 6 U.S. President’s Malaria Initiative, USAID, Conakry, Guinea

**Keywords:** Stock-out, Artemisinin-based combination therapy, Rapid diagnostic test, Logistics management information system, Data quality

## Abstract

**Background:**

Ensuring malaria commodity availability at health facilities is a cornerstone of malaria control. Since 2013, the Guinea National Malaria Control Programme has been routinely collecting data on stock levels of key malaria commodities through a monthly routine malaria information system (RMIS). In parallel, biannual end-user verification (EUV) surveys have also assessed malaria commodity availability at a subset of health facilities, potentially representing a duplication of efforts.

**Methods:**

Data on 12 malaria commodity stock levels verified during four EUV surveys conducted between 2014 and 2016 was compared to data for the corresponding months submitted by the same health facilities through the RMIS. The sensitivity and specificity of the RMIS in detecting stock-outs was calculated, as was the percent difference between average stock levels reported through the two systems.

**Results:**

Of the 171 health facilities visited during the four EUV surveys, 129 (75%) had data available in the RMIS. Of 351 commodity stock-outs observed during the EUV in the sampled reporting health facilities, 256 (73%) were also signaled through the corresponding RMIS reports. When the presence of malaria commodity stocks was confirmed during the EUV surveys, the RMIS also reported available stock 87% (677/775) of the time. For all commodities, the median percent difference in average stock levels between the EUV and RMIS was 4% (interquartile range − 7 to 27%).

**Conclusion:**

The concordance between stock levels reported through the RMIS and those verified during the EUV visits provides certain evidence that RMIS data can inform quantification and procurement decisions. However, lower than acceptable rates of reporting and incomplete detection of stock-outs from facilities that do report suggest that further systems strengthening is needed to improve RMIS reporting completeness and data quality.

## Background

Malaria transmission occurs year-round throughout Guinea and is the principal cause of healthcare seeking in the public sector, responsible for 31% of all outpatient consults [1]. The country has made substantial progress in malaria control in recent years. The national prevalence of malaria infection in children under 5 years of age, as measured by microscopy, fell from 44% in 2012 [2] to 15% in 2016 [3]. Malaria incidence in 2016 was estimated to be 87/1000 population by the Guinea National Malaria Control Programme (NMCP) [4].

The Guinea NMCP aims to reduce malaria morbidity and mortality through a combination of interventions aimed at prevention, consisting of the distribution of long-lasting insecticidal nets (LLINs), seasonal malaria chemoprevention, and intermittent preventive treatment of malaria during pregnancy, as well as interventions to ensure timely diagnosis and correct treatment of confirmed malaria cases. Ensuring effective treatment requires a steady and reliable supply of malaria tests and medicines at health facilities.

Key malaria commodities include malaria rapid diagnostic tests (RDTs), medicines used for treatment of uncomplicated malaria by artemisinin-based combination therapy (ACT), quinine tablets, injectable forms of quinine and artemisinin for treatment of severe malaria, sulfadoxine–pyrimethamine (SP) for intermittent preventive treatment in pregnancy, and LLINs for routine distribution to pregnant women and infants. These commodities are financed and centrally procured by the government of Guinea and donors, primarily the Global Fund to Fight AIDS, Tuberculosis and Malaria (GF) and the U.S. President’s Malaria Initiative (PMI), and stored at the central medical stores. From there, they are distributed to regional depots, from which Guinea’s roughly 400 public hospitals and health centres are supplied when they directly request commodities from the regional depot based on needs, using a “pull system”. Health centres in turn supply the community health workers and health posts in their catchment area. Malaria commodities are provided free of charge to patients of all ages.

Monitoring stocks of malaria commodities throughout the system is a priority activity for the NMCP and its partners. Stock availability is an important indicator of health system readiness and sudden interruptions in the supply chain may affect the quality of service and health programmes [5]. Moreover, frequent stock-outs of essential anti-malarial drugs due to improperly managed supply chains can affect healthcare provider prescription behavior [6], increase patient healthcare costs [7], and impede universal access to quality malaria case management [5, 8, 9]. However, while there has been work on developing tools for malaria supply chain management in endemic countries [10], few formal studies of optimal supply chain policies have been performed [11–13].

During initial expansion of malaria control efforts in Guinea, the country’s logistic management and information system (LMIS) at the time was originally targeted to manage and improve management of commodities at the level of the central medical stores and regional depots. Because the LMIS was not designed to collect data on commodity stocks at the health-facility level, the Guinea NMCP decided to include data on malaria commodity stocks as part of its monthly routine malaria information system (RMIS), parallel to and independent of the larger health management information system. A parallel malaria-only reporting system was deemed necessary in the absence of a functional health management information system at the time. The RMIS is supported by PMI and GF and was rolled out in 2013, reaching nationwide coverage in 2014. District-level reporting improved from 66% completeness in late 2014, shortly after expansion, to consistent 100% completeness since early 2016. Data on stock levels at the beginning of the month, stock levels at the end of the month, number of days of stock-out, and consumption rates, including use, expiration, and loss, are reported through the RMIS by hospitals and health centres for 12 malaria commodities. Monthly data are recorded in paper forms at health facilities, and then transferred to the district health authorities, where they are inputted into an electronic database and sent to the national level. The Guinea NMCP routinely analyses the reported data to identify areas with under- and over-stock of commodities and monitors stock levels and consumption rates to forecast commodity needs. The malaria monthly bulletin issued by NMCP has included district-level commodity indicators since its inception in late 2014 and is a key tool in allowing NMCP and its partners to track malaria commodity levels.

When PMI was launched in Guinea in 2012 and the RMIS was not yet operational, PMI implemented periodic end-use verification (EUV) surveys to monitor malaria commodity availability at regional warehouses and in peripheral health facilities [14]. Originally, the EUV surveys were only implemented in the 19 health districts supported by PMI, but starting July 2014, implementation expanded nationwide. During these surveys, teams from the national level visited a cross-sectional sample of hospitals and health centres to record stocks of malaria commodities, examine stock cards, document storage conditions, and review outpatient registries. The surveys were implemented by the PMI-funded Systems for Improved Access to Pharmaceuticals and Services project, but included participation from NMCP and district health authorities. They followed a standard methodology to track PMI-procured commodities from source to end-user.

Since 2016, the improved timeliness and completeness of the RMIS have raised the possibility that it might already be collecting sufficiently reliable commodity data to provide the same kind of accountability that the EUV surveys were originally designed to provide. If that were the case, then the resources currently devoted to the EUV surveys could be redirected to other monitoring and evaluation activities. To explore this possibility, NMCP and its partners undertook a systematic assessment comparing the RMIS commodity reporting to the results of the EUV.

## Methods

A retrospective analysis was conducted comparing data on commodity stock-outs and stock levels reported through the RMIS to data collected in health facilities during four EUV surveys. Using the EUV data as the gold standard, the RMIS data were evaluated on reporting completeness, sensitivity and specificity of stock-out detection, and the relative difference in reported stock levels. The RMIS data were obtained from the NMCP national malaria database, and the EUV data were provided by the PMI-funded Systems for Improved Access to Pharmaceuticals and Services project in Guinea.

### Period and area

Four EUV surveys that occurred since the scale-up of the RMIS were included in the analysis: July 2014, December 2014, October 2015, and August 2016. The number of health facilities visited during the surveys varied from 31 in the two 2014 surveys to 64 in the most recent survey in August 2016. In total, the four surveys comprised 171 hospital and health centre visits throughout Guinea; 71 (42%) of these were to visits to health facilities visited more than once during the four rounds. Hospitals represented 19% of all health facility visits across the four surveys. The number of regions visited during the surveys was six in July 2014, increasing to seven in December 2014, and finally including all eight regions in the October 2015 and August 2016 surveys.

### Data analysis

For each health facility visited during the EUV surveys, the RMIS database was searched by matching on health facility name and district. Variations in spelling and health facility name required manual matching of health facilities in the two databases. Data on day-of-visit commodity stock levels verified during the surveys were abstracted from the EUV database. Data on beginning-of-month and end-of-month stock levels and the number of days with stock-out were abstracted from the RMIS for the month corresponding to the EUV visit for each health facility in the EUV. Data on twelve malaria commodities were analysed: RDTs, the four age/weight formulations of amodiaquine–artesunate (AS–AQ), oral quinine tablets, injectable quinine, injectable artesunate, intramuscular artemether, SP, and LLINs. Data on stocks of AL, which was not in wide use in Guinea in the period covered by the assessment, were considered separately. The July 2014 and December 2014 EUV surveys did not capture data on AL, so indicators for AL for these surveys were not calculated. Moreover, in the period covered by the four surveys, the RMIS data recording and reporting forms did not stratify AL stocks by its four age/weight formulations, so data on AL stocks collected during the EUV surveys, which did stratify by the different formulations, were considered together; AL was considered in stock if any formulation of AL was documented, and AL stock levels were considered to be the sum across all AL formulations.

Reporting completeness of the RMIS was assessed by calculating the proportion of the health facilities included in each EUV survey that had submitted their monthly malaria report through the RMIS for the month of the survey.

The sensitivity and specificity of the RMIS to detect stock-outs of malaria commodities were also assessed. Sensitivity was calculated as the proportion of health facilities with a stock-out during the EUV survey that also reported a stock-out in the RMIS report for the corresponding month. The specificity was defined as the proportion of health facilities without a stock-out during the EUV survey that did not report a stock-out in their corresponding RMIS report. Health facilities that had not submitted RMIS data during the month of the survey were excluded from the analysis of specificity and sensitivity. Stock-outs in the EUV were defined as zero stock level recorded during the day of the survey visit. In the RMIS data, a health facility was considered to have reported a stock-out if beginning-of-month stock levels were reported as zero, end-of-month stock levels were reported as zero, or the number of days of stock-out during the month were greater than zero. The sensitivity and specificity were calculated separately for each commodity and each survey.

The stock levels reported through the RMIS and counted during the EUV visits were compared separately for each survey and then jointly across all surveys. Because day-of-visit data were not available from the RMIS, the average monthly stock levels were analysed, defined as the mean of the beginning-of-month and end-of-month stock levels. For each commodity, the total mean monthly stock level per health facility was calculated for the RMIS dataset, and the average percent difference between the RMIS and EUV datasets was calculated. Additionally, for each individual health facility, the percent difference between the EUV and RMIS data was calculated for each commodity. The distribution of health-facility-level percent difference was visualized using boxplots, stratifying by commodity. The proportion of health facilities with percent difference < 25%, 25–50%, and > 50% was calculated. Health facilities that had not submitted RMIS data during the month of the survey were excluded from the analysis of stock levels.

Abstracted data were collected in an Excel (Microsoft, Redmond, USA) spreadsheet and data analysis was performed using R version 3.3.2 (R Foundation for Statistical Computing, Vienna, Austria).

## Results

### Completeness of health facility reporting

Out of the 171 health facilities visited during the four EUVs, 75% (129/171) had submitted corresponding monthly reports through the RMIS (Table 1). Completeness ranged from 67% (30/45) in the October 2015 survey to 87% (27/31) in the July 2014 survey.

**Table 1:** Proportion of health facilities visited during four end-user verification (EUV) surveys that also submitted reports through the routine malaria information system, Guinea

Reporting completeness									
EUV Jul 2014		EUV Dec 2014		EUV Oct 2015		EUV Aug 2016		Total	
n/N	%	n/N	%	n/N	%	n/N	%	n/N	%
27/31	87	24/31	77	30/45	67	48/64	75	129/171	75

### Stock-out detection

There were 351 instances of stock-outs for the 12 malaria commodities assessed recorded during the four EUV surveys in health facilities that had submitted RMIS reports. Of these commodity stock-outs, 256 (73%) were also signaled through the corresponding RMIS reports (Table 2). The sensitivity of the RMIS in detecting stock-outs of all commodities ranged from 68% (119/176) in the August 2016 survey to 84% (53/63) in the July 2014 survey. Across all surveys, sensitivity of the RMIS was highest in detecting injectable artemether stock-outs (sensitivity 98%, 51/52) and least sensitive for SP (sensitivity 31%, 5/16). The RMIS was also highly specific in capturing stock availability. For facilities visited during the EUV for which there was a corresponding RMIS report, there were 775 instances across all commodities and all four surveys where the EUV reported available stock. In the RMIS database, 677 (87%) of these instances were reported having stock available during the corresponding months. The specificity for all commodities ranged from 84% (173/205) in October 2015 to 91% (130/143) in December 2014. The RMIS was most specific for detecting stock availability of the ASAQ formulations and AL, with specificity exceeding 88% for each. Specificity was lowest for injectable artemether, 43% (6/14).

**Table 2:** Sensitivity and specificity of the routine malaria information system (RMIS) in detecting stock-outs observed during four end-user verification (EUV) surveys, Guinea

Commodity	EUV Jul 2014		EUV Dec 2014		EUV Oct 2015		EUV Aug 2016		Total	
	Sensitivity^a^	Specificity^b^	Sensitivity^a^	Specificity^b^	Sensitivity^a^	Specificity^b^	Sensitivity^a^	Specificity^b^	Sensitivity^a^	Specificity^b^
RDT	4/4 (100%)	20/21 (95%)	1/3 (33%)	17/17 (100%)	4/5 (80%)	16/24 (67%)	7/15 (47%)	24/27 (89%)	16/27 (59%)	77/89 (87%)
ASAQ infant	2/4 (50%)	19/21 (90%)	0/1 (0%)	19/20 (95%)	0/2 (0%)	21/21 (100%)	12/16 (75%)	21/26 (81%)	14/23 (61%)	80/88 (91%)
ASAQ small child	1/2 (50%)	20/23 (87%)	–	20/20 (100%)	–	21/23 (91%)	9/17 (53%)	23/26 (88%)	10/19 (53%)	84/92 (91%)
ASAQ child	1/2 (50%)	21/23 (91%)	0/2 (0%)	17/19 (89%)	–	22/23 (96%)	4/10 (40%)	28/30 (93%)	5/14 (36%)	88/95 (93%)
ASAQ adult	1/3 (33%)	17/22 (77%)	2/4 (50%)	15/17 (88%)	1/1 (100%)	20/23 (87%)	5/5 (100%)	32/34 (94%)	9/13 (69%)	84/96 (88%)
AL	–	–	–	–	–	5/5 (100%)	13/14 (93%)	8/8 (100%)	13/14 (93%)	13/13 (100%)
SP	2/4 (50%)	19/20 (95%)	0/2 (0%)	15/16 (94%)	2/3 (67%)	24/24 (100%)	1/7 (14%)	30/34 (88%)	5/16 (31%)	88/94 (94%)
Injectable artesunate	15/15 (100%)	0/1 (0%)	15/15 (100%)	5/5 (100%)	1/3 (33%)	2/3 (67%)	10/12 (83%)	0/1 (0%)	41/45 (91%)	7/10 (70%)
Injectable artemether	5/5 (100%)	–	19/20 (95%)	0/1 (0%)	1/1 (100%)	2/6 (33%)	26/26 (100%)	4/7 (57%)	51/52 (98%)	6/14 (43%)
Injectable quinine	1/1 (100%)	23/24 (96%)	3/5 (60%)	14/16 (88%)	6/9 (67%)	12/17 (71%)	15/21 (71%)	13/17 (76%)	25/36 (69%)	62/74 (84%)
Quinine tablet	13/15 (87%)	5/9 (56%)	14/16 (88%)	3/4 (75%)	9/10 (90%)	10/16 (62%)	3/15 (20%)	23/27 (85%)	39/56 (70%)	41/56 (73%)
LLIN	8/8 (100%)	8/8 (100%)	5/9 (56%)	5/8 (62%)	1/1 (100%)	18/20 (90%)	14/18 (78%)	16/18 (89%)	28/36 (78%)	47/54 (87%)
All commodities	53/63 (84%)	152/172 (88%)	59/77 (77%)	130/143 (91%)	25/35 (71%)	173/205 (84%)	119/176 (68%)	222/255 (87%)	256/351 (73%)	677/775 (87%)

*RDT* Rapid diagnostic test, *ASAQ* artesunate–amodiaquine, *AL* artemether–lumefantrine, *SP* sulfadoxine–pyrimethamine, *LLIN* long-lasting insecticidal net

^a^ Proportion of health facilities without stock during the EUV survey that had also reported a stock-out in their RMIS report

^b^ Proportion of health facilities that were not stocked-out during the EUV survey that had not reported a stock-out in their RMIS report

### Stock levels

**Fig. 1:**
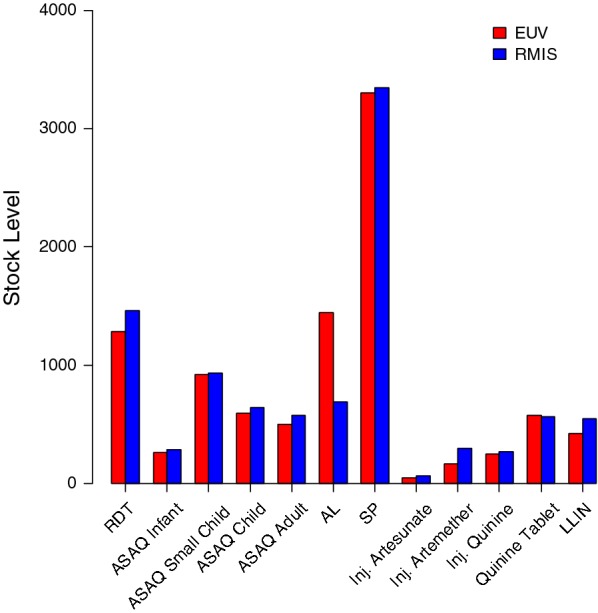
Average stock levels (individual commodity units) per health facility for 12 malaria commodities reported through the Guinea routine malaria information system (RMIS) and verified during four end-user verification (EUV) surveys conducted between 2014 and 2016, averaged across all four surveys, for the subset of health facilities with data available in both data sources. *RDT* Rapid diagnostic test, *ASAQ* artesunate–amodiaquine, *AL* artemether–lumefantrine, *SP* sulfadoxine–pyrimethamine, *LLIN* long-lasting insecticidal net

**Fig. 2:**
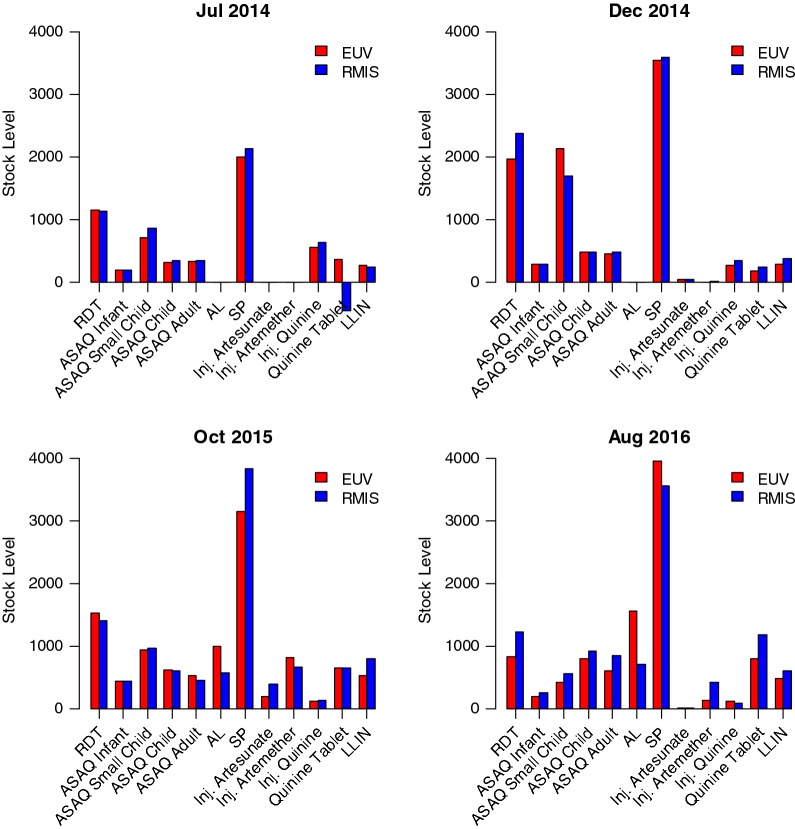
Average stock levels (individual commodity units) per health facility for 12 malaria commodities reported through the Guinea routine malaria information system (RMIS) and verified during four end-user verification (EUV) surveys for the subset of health facilities with data available in both data sources. *RDT* Rapid diagnostic test, *ASAQ* artesunate–amodiaquine, *AL* artemether–lumefantrine, *SP* sulfadoxine–pyrimethamine, *LLIN* long-lasting insecticidal net

**Fig. 3:**
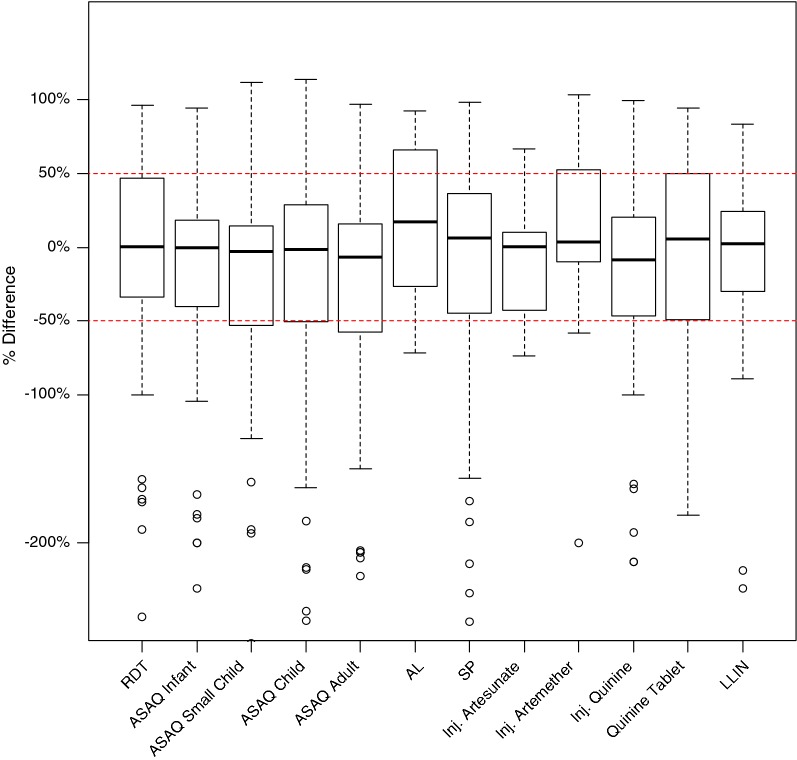
Distribution of percent differences between stock levels reported through the routine malaria information system and those verified during four end-user verification surveys, averaged across all four surveys, Guinea. *RDT* Rapid diagnostic test, *ASAQ* artesunate–amodiaquine, *AL* artemether–lumefantrine, *SP* sulfadoxine–pyrimethamine, *LLIN* long-lasting insecticidal net. Boxes show median values and interquartile ranges. Whiskers show extreme values up to 1.5 times interquartile range and circles represent outliers outside this range

Average commodity stock levels reported through the RMIS were close to the average stock levels verified during the EUV surveys (Figs. 1 and 2). The median percent difference in average stock levels between the EUV and RMIS across all commodities in the four surveys was 4%, with the interquartile range spanning − 7% to 27% (Fig. 3).

**Fig. 4:**
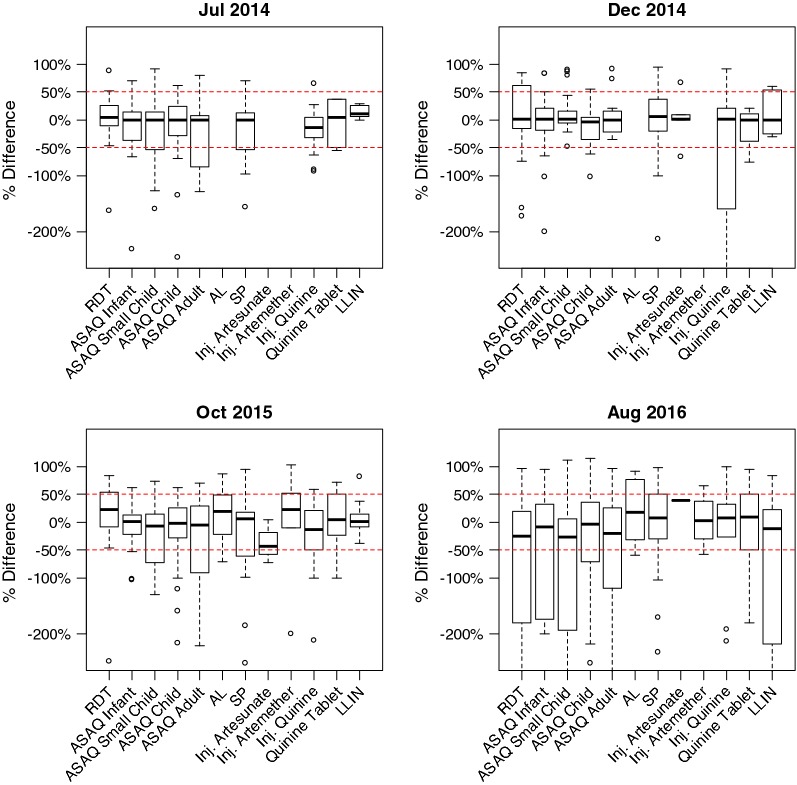
Distribution of percent differences between stock levels reported through the routine malaria information system and those verified during four end-user verification surveys, Guinea. *RDT* Rapid diagnostic test, *ASAQ* artesunate–amodiaquine, *AL* artemether–lumefantrine, *SP* sulfadoxine–pyrimethamine, *LLIN* long-lasting insecticidal net. Boxes show median values and interquartile ranges. Whiskers show extreme values up to 1.5 times interquartile range and circles represent outliers outside this range

The concordance between average stock levels across the two data sources belied substantial variation at the individual health facility level (Fig. 3). However, the median percent difference was close to zero for all commodities, and, for all commodities with the exception of injectable artemether, more than half of all absolute percent differences were less than 50% (Table 3). The distribution of percent difference was stable across the four surveys (Fig. 4).

**Table 3:** Distribution of percent differences between malaria commodity stock levels reported through the routine malaria information system compared to those verified during four end-user verification surveys, Guinea

Commodity	Number of reports		
	< 25% difference	25–50% difference	> 50% difference
RDT	37/92 (40%)	15/92 (16%)	40/92 (43%)
ASAQ infant	47/93 (51%)	11/93 (12%)	35/93 (38%)
ASAQ small child	47/99 (47%)	13/99 (13%)	39/99 (39%)
ASAQ child	44/102 (43%)	17/102 (17%)	41/102 (40%)
ASAQ adult	40/98 (41%)	22/98 (22%)	36/98 (37%)
AL	4/15 (27%)	4/15 (27%)	7/15 (47%)
SP	40/98 (41%)	17/98 (17%)	41/98 (42%)
Injectable artesunate	4/9 (44%)	2/9 (22%)	3/9 (33%)
Injectable artemether	4/10 (40%)	0/10 (0%)	6/10 (60%)
Injectable quinine	31/74 (42%)	18/74 (24%)	25/74 (34%)
Quinine tablets	20/53 (38%)	6/53 (11%)	27/53 (51%)
LLIN	25/53 (47%)	11/53 (21%)	17/53 (32%)

*RDT* Rapid diagnostic test, *ASAQ* artesunate–amodiaquine, *AL* artemether–lumefantrine, *SP* sulfadoxine–pyrimethamine, *LLIN* long-lasting insecticidal net

## Discussion

The RMIS data on overall malaria commodity stock levels generally agreed with the stock levels counted during the field visits. However, the detection of stock-outs through the RMIS is still incomplete. About a quarter of health facilities do not report through RMIS, and in the three quarters of health facilities that had submitted malaria reports for the months of the EUV visits, the RMIS was sufficiently sensitive to detect only three quarters of all stock-outs observed in the EUV. The RMIS reported stock-outs not captured by the EUV in 13% of cases.

The concordance between the two data sources should be interpreted in the context of the limitations of the methodology of the comparison. Because the EUV surveys only record the actual stock levels on the day of the health facility visit, the data from the monthly RMIS reports and the EUV data could not be compared directly. As a result, the definition of stock-outs differed between the two data sources, and the sensitivity and specificity reported here need to be interpreted in that context. In particular, the sensitivity could have been overestimated and the specificity underestimated since the RMIS can capture stock-outs that occur at any point during the month. In addition, the comparison of stock levels was not a one-to-one comparison, which could account for the observation that although mean stock levels were similar between the two sources, there was substantial variation at the individual health facility level. A further limitation is that there were differences in how AL stock levels were captured in the two systems. Finally, non-reporting health facilities might be more likely to have stock-outs or poor data recording practices, and thus the concordance between the EUV and RMIS stock levels reported here might be an over-estimate.

Ultimately, the complexities in validating the RMIS data against the EUV data reflect fundamental differences in the reporting systems’ objectives and designs. The RMIS was designed to provide complete, timely data on key health facility malaria indicators for the entire country throughout the year. In contrast, EUV surveys were designed to provide periodic audits of health facilities for the purposes of accountability. The imperfect data completeness, sensitivity and specificity of the RMIS are balanced out by a much larger amount of data collected during the RMIS. For 2016, while the EUV survey visited a total of 64 health facilities, yielding 768 data-points on stock availability, the RMIS collected a total of 5280 monthly health facility reports across the entire year, for a total of 63,360 data points. The spatial and temporal scope of the RMIS allows continuous detection of stock-outs throughout the country. Similarly, the representativeness of the consumption and stock level data in the RMIS allows for accurate quantification and forecasting of malaria commodity needs. Finally, the fact that the RMIS is managed and implemented by the NMCP and district health authorities means that it is sustainable in the long term and permits the kind of routine analysis embodied by the production and dissemination of the monthly malaria bulletins.

Because it involves teams visiting health facilities, the EUV methodology allows for the incidental collection of data that are not normally available through the RMIS. Verification of commodity stock by survey teams allows for independent assessment of the availability of donor-sourced malaria commodities. In addition, survey teams assess other aspects of management of malaria commodities, such as storage conditions and the correct use of stock cards. EUV surveys in Guinea also include a retrospective review of outpatient registries, providing insight into malaria case management practices. Finally, EUVs provide a chance for capacity building for participating NMCP and district health authority staff, while also providing an incidental opportunity for supportive supervision of health facility staff.

The results presented here suggest that the data reported through the Guinea RMIS could be used to monitor commodity stocks in health facilities. In this context, the EUVs do not provide substantial additional actionable information on malaria commodity stock levels and the stock verification component of the EUVs might represent an unnecessary duplication of efforts, on the condition that the RMIS can routinely be queried to provide data on commodity stock levels and stock-outs. Instead, the added utility of the EUV surveys is concentrated in the secondary data incidentally collected during the surveys; for example, storage conditions and case management practices and their role in supervision and capacity building. In recent years, the Guinea NMCP has developed and rolled out a comprehensive supervision tool to be used during malaria-related supervisory visits to health facilities. The additional components of the EUV, such as those related to storage conditions and commodity management practices, are either already included in the supervision tool or could be easily integrated into the existing tool. Resources currently devoted to the implementation of the EUV survey could instead be repositioned to support routine supervisory visits, including increasing their frequency, expanding national- and district-level participation, and enhancing their focus on data quality. As malaria control efforts in Guinea mature, the focus of NMCP and its partners has been shifting from scale-up of interventions to monitoring and tracking their impact. In parallel, the periodic collection of data through household and health facility surveys has yielded to continuous, routine information systems.

## Conclusions

Discontinuation of surveys, originally intended to be temporary measures in the absence of well-functioning routine systems, will need to be informed by systematic evaluations like the one reported here that can assess whether the sensitivity and specificity of routine information systems are adequate and can provide evidence that the routine information systems are sufficiently robust and accurate. Ultimately, the assessment of the commodity data in the RMIS reported here was made possible because of the unique opportunity to have an independent dataset on commodity stocks in the form of the EUV surveys. The ability to rely on the RMIS to provide high-quality commodity data will benefit from future assessments that cross-check independently collected data, such as from data quality audits, with the routinely collected RMIS data. These sorts of assessments are particularly timely as Guinea pursues a modernization of its ensemble HMIS, based on the increasingly popular open-source District Health Information System 2 (DHIS2) reporting platform. This new system has an integrated health-facility-level LMIS, whose framework is modeled on the commodity component of the RMIS. Eventually, the RMIS itself is planned to be fully integrated into the new HMIS, cementing it as a foundation of routine disease surveillance in Guinea.
